# Thirty Years of Sweat Chloride Testing at One Referral Center

**DOI:** 10.3389/fped.2017.00222

**Published:** 2017-10-26

**Authors:** Alethéa Guimarães Faria, Fernando Augusto Lima Marson, Carla Cristina Souza Gomez, Maria de Fátima Servidoni, Antônio Fernando Ribeiro, José Dirceu Ribeiro

**Affiliations:** ^1^Department of Pediatrics, School of Medical Sciences, University of Campinas, Campinas, Brazil; ^2^Laboratory of Pulmonary Physiology, Center for Pediatrics Investigation, School of Medical Sciences, University of Campinas, Campinas, Brazil; ^3^Department of Medical Genetics, School of Medical Sciences, University of Campinas, Campinas, Brazil

**Keywords:** cystic fibrosis, diagnosis, sweat chloride, sweat sodium, sweat test

## Abstract

**Objective:**

To conduct a descriptive analysis of the sweat test (ST), associating ST results with epidemiological data, *CFTR* (cystic fibrosis transmembrane conductance regulator) mutations and reasons to indicate the ST, as well as correlating sweat sodium and sweat chloride concentrations in subjects.

**Methods:**

Retrospective survey and descriptive analysis of 5,721 ST at a university referral center.

**Results:**

The inclusion of the subjects was based on clinical data related with cystic fibrosis (CF) phenotype. The samples were grouped by (i) sweat chloride concentrations (mEq/L): <30: 3,249/5,277 (61.6%); ≥30 to <60: 1,326/5,277 (25.1%); ≥60: 702/5,277 (13.3%) and (ii) age: (Group A––GA) 0 to <6 months; (Group B––GB) ≥6 months to <18 years; (Group C––GC) ≥18 years. Digestive symptoms showed higher prevalence ratio for the CF diagnosis as well as association between younger age and higher values of sweat chloride, sweat sodium, and chloride/sodium ratio. The indication of ST due to respiratory symptoms was higher in GB and associated with greater age, lower values of sweat chloride, sweat sodium, and chloride/sodium ratio. There was higher prevalence of ST with sweat chloride levels <30 mEq/L in GB, ≥60 mEq/L in GC, and with borderline level in GB. There was positive correlation between sweat sodium and sweat chloride. Sweat chloride/sweat sodium and sweat sodium–sweat chloride indexes showed association with sex, reason for ST indication, and *CFTR* mutations. Sex alters some values presented in the ST. The number of ST/year performed before and after the newborn screening implementation was the same; however, we observed a higher number of borderlines values. A wide spectrum of *CFTR* mutation was found. Severe *CFTR* mutations and F508del/F508del genotype were associated with highest probability of ST chloride levels ≥60 mEq/L, and the absence of *CFTR* mutations identified was associated with borderline ST and respiratory symptoms.

**Conclusions:**

ST data showed wide variability dependent on age, sex, reason for examination indication, *CFTR* mutations, and weight of the collected sweat sample. Sweat sodium concentration is directly correlated with sweat chloride levels and it could be used as a quality parameter.

## Introduction

Cystic fibrosis (CF) (OMIM: #219700) is a chronic disease that leads to variability of genotypic and phenotypic expression. The CF diagnosis is based on neonatal screening findings and/or phenotypic manifestations, family history, and higher chloride ion (Cl^−^) concentration in sweat, in addition to two mutations in the *CFTR* gene (cystic fibrosis transmembrane conductance regulator) (OMIM: *602421) (1). If mutations in the *CFTR* gene and/or altered CFTR protein functions cannot be detected by any method, a definitive CF diagnosis cannot be made.

The function and/or presence of the CFTR protein has been demonstrated in the sweat glands by measurement of ion concentrations in sweat [sweat test (ST) and evaporimeter] (2–5), nasal epithelium (nasal potential difference) (6), salivary gland (ions in saliva) (7), and in the digestive tract (presence and function of the CFTR protein in rectal biopsy) (8, 9). Although *CFTR* gene mutations are the most important and appropriate markers for CF diagnosis, the analysis of ion chloride concentration in sweat is still considered the gold standard for the diagnosis, as well as the simplest method used to assess functional properties of the CFTR protein. In 1938, Dr. Dorothy Hansine Andersen pointed out that the high concentration of salt in sweat of patients with CF was almost accepted as pathognomonic for CF (10).

Over the past 65 years, there have been advances in the implementation and interpretation of the ST (3). Sweat chloride concentrations ≥60 mEq/L in at least two tests performed at different collection times are considered the gold standard for CF diagnosis. Individuals with borderline values ranging from 30 to 59 mEq/L require evaluation of the mutations in the *CFTR* gene (11).

The application of appropriate methods to perform ST is essential for accurate CF diagnosis. Therefore, referral centers should follow internal procedures which are in line with the guidelines provided by the cystic fibrosis foundation (CFF) (12). One of the ST collection methods approved by the CFF was developed by Gibson and Cooke (2). The quantitative pilocarpine iontophoresis ST determines the weight of sweat collected with analytical balance. Special care should be taken to avoid evaporation of the sample. This procedure is likely to fail unless it is carried out by experienced and trained personnel (13).

Currently, sweat chloride concentration dosage has also been useful to demonstrate the function of the CFTR protein after the administration of correctors, potentiators, or stabilizers drugs by personalized/precision medicine (14). Therefore, the future role of ST should include the successful monitoring of personalized medicine therapy.

The aim of this study was to conduct a descriptive analysis of the ST, associating ST results with epidemiological data, *CFTR* mutations screening, and reasons to indicate the test (respiratory, digestive, or nutritional symptoms), as well as correlating concentrations of sweat sodium and sweat chloride in subjects.

## Materials and Methods

A retrospective study between 1986 and 2016, characterizing 30 years of monitoring, was conducted on 5,721 sweat samples at a CF reference center.

The sweat was collected following the traditional Gibson–Cooke method (2), and concentrations of sweat chloride and sweat sodium were determined by chloridometry and flame photometry, respectively.

This study was carried out in accordance with the recommendations of Ethics Committee of the University of Campinas (Protocol no. 474326), with written informed consent from the institution in accordance with the Declaration of Helsinki. The ST and subjects’ clinical data were obtained from medical records of the Pediatric Gastroenterology Laboratory, and from the Gastroenterology Center at the University Hospital, namely, name, subject’s age at the time of the examination, sex, indication for the ST (respiratory, digestive, or nutritional symptoms), *CFTR* mutation screening, immunoreactive trypsinogen (IRT) inclusion in our center, weight of the sweat sample collected, sweat chloride and sweat sodium concentrations, sweat chloride/sweat sodium ratio, and sweat sodium–sweat chloride in the ST samples. Samples were excluded according to the following criteria: sweat weight <75 mg and >400 mg, sweat chloride level ≤10 mEq/L or >160 mEq/L, sweat sodium level ≤10 mEq/L or >150 mEq/L, or absence of descriptive data (sweat weight and sweat sodium concentrations).

The age of patients was divided into three groups: (i) from birth to <6 months; (ii) ≥6 months to <18 years; (iii) ≥18 years (11, 15). The sweat chloride levels were used to group the samples according to the CF diagnosis: (i) chloride <30 mEq/L; (ii) chloride ≥30 to <60 mEq/L; (iii) chloride ≥60 mEq/L (group of patients with CF) (11).

All requested tests have been analyzed. For some subjects, the test involved more than one sweat sample.

### Identification of Mutations in the *CFTR* Gene

*CFTR* mutations were analyzed by polymerase chain reaction techniques for F508del and enzymatic digestion for G542X, R1162X, R553X, G551D, and N1303K. Other mutations in *CFTR* were also identified by sequencing or using the SALSA MLPA technique (multiplex ligation-dependent probe amplification) Kit P091-C1 CFTR-MRC-Holland: S4X, 2183 A > G, 1717-G > A, I618T, with MegaBace1000^®^ (GE Healthcare Biosciences, Pittsburgh, PA, USA) and ABI 3500 (Applied Biosystems-Thermo Fisher Scientific, São Paulo, Brazil) (16).

Considering the *CFTR* genotypes, patients were divided into three groups: (i) with two identified mutations belonging to class I, II, and/or III; (ii) with one identified mutation belonging to class I, II, and/or III; (iii) no identified mutation belonging to class I, II, and/or III. Other identified mutations in the *CFTR* gene, belonging to class IV, V, and/or VI, were not included in the statistical analysis. The *CFTR* mutation classification is according to the literature (17).

### Statistical Analysis

A descriptive analysis was used with a number of observations, mean value, standard deviation, median, and minimum and maximum values for continuous variables. Confidence interval (95%) was calculated for proportions. For categorical variables, the data are presented by frequencies and percentages.

Statistical analyses were performed using the Statistical Package for the Social Sciences software version 23.0 (IBM Corp. Released 2015. IBM SPSS Statistics for Windows, Version 23.0. Armonk, NY: IBM Corp.) and OpenEpi version 3.03a. The comparison between the variables with categorical distribution was carried out by chi-square test and Fisher’s exact test, depending on the data distribution. Mann–Whitney and Kruskal–Wallis non-parametric tests were used for the analysis of variables with numerical distribution. Spearman’s rank correlation test and linear regression were used to compare the association between two variables with numerical distribution. Significance level was set at 0.05 for all analyses. The level of power in sample size calculations was >80%.

## Results

This study assessed 5,721 sweat samples of STs that had been requested due to clinical presentations compatible with CF and/or neonatal screening with abnormal IRT values. One excluded 444 (7.78%) of these sweat samples, as follows: (i) 23 showed sweat weight <75 mg; (ii) four lacked sweat weight described in the examination; (iii) one lacked laboratory data; (iv) one showed no sweat sodium value described in the examination; (v) 395 showed sweat chloride level <10 mEq/L; (vi) nine showed sweat chloride level >160 mEq/L; (vii) four showed sweat sodium level <10 mEq/L; (viii) one showed sweat sodium level >150 mEq/L; (ix) six showed sweat weight >400 mg. Thus, the final study included 5,277 sweat samples. Sex of 15 (0.28%) subjects was not obtained because the examination was carried out on the newborn’s mother’s name after neonatal screening. Thus, 2,786/5,262 (52.9%) samples of male subjects were included and analyzed.

The IRT inclusion in our center was dated since 2010. From our subjects, 4,020/5,265 (76.4%) were included before 2010 and 1,245/5,265 (23.6%) after this date. In this context, there were performed 174.78 and 177.86 ST/year on average before and after IRT inclusion, respectively.

The STs were carried out at an outpatient clinic which is primarily intended for individuals under 25 years of age. Therefore, only a few examinations were performed in adult individuals. This explains the low inclusion rate of adults compared with children and teenagers.

The mean age of subjects was 12.84 ± 18.28 years; median of five (ranging from 0 to 85.58) years. However, the age in 80/5,277 (1.5%) samples was not entered at the time of the examination. Thus, three age groups were built with the following frequencies: (i) birth to six months: 567/5,197 (10.9%) samples; (ii) ≥6 months to <18 years: 3,558/5,197 (68.5%) samples; (iii) ≥18 years: 1,072/5,197 (20.6%) samples. Moreover, in 61/5277 samples, we had the age groups without the exact age by years and/or months.

The mean level of sweat chloride was 34.01 ± 27.16 mEq/L, median of 23.74 (ranging from 10 to 159.2) mEq/L. The mean level of sweat sodium was 37.76 ± 21.31 mEq/L, median of 30.9 (ranging from 10 to 149.7) mEq/L. Subjects were referred to the ST due to the following symptoms (each symptom was analyzed separately): (i) respiratory symptoms: 2,623/3,400 (77.1%); (ii) digestive symptoms: 419/3,400 (12.3%); (iii) nutritional symptoms: 391/3,400 (11.5%). In addition, we analyzed the presence of simultaneous digestive and respiratory symptoms, and we observed (i) no symptom––577/3,400 (17%); (ii) one symptom––2,604/3,400 (76.6%); (iii) two symptoms––219/3,400 (6.4%).

Initial ST request information could not be obtained for 1,877/5,277 (35.57%) sweat samples. The chloride–sodium ratio showed mean level of 0.84 ± 0.23 mEq/L; median of 0.82 mEq/L (ranging from 0.29 to 2.31 mEq/L). The sodium–chloride difference showed mean level of 3.75 ± 10.38 mEq/L; median of 5.3 mEq/L (ranging from −82.7 to 42.4 mEq/L). The weight of the sweat samples showed mean level of 170 ± 40 mg and median of 170 mg (ranging from 80 to 370 mg).

The sweat samples were divided into three groups of sweat chloride levels: (i) <30 mEq/L: 3,249/5,277 (61.6%); (ii) ≥30 to <60 mEq/L: 1,326/5,277 (25.1%); (iii) ≥60 mEq/L: 702/5,277 (13.3%).

Table 1 shows the association between sweat chloride value, sex of the subject, and reason for the indication of the examination. The indication due to nutritional symptoms showed no positive association (*p* = 0.678). The indication due to respiratory reasons showed no correlation with the CF diagnosis, unlike the indication due to digestive reasons. In the ST, for sweat chloride, males had lower prevalence ratio for borderline values and values ≥60 mEq/L, as well as higher prevalence ratio for values <30 mEq/L.

**Table 1:** Association between sweat chloride levels, sex of the subject, and reason for indication of sweat test.

Chloride levels (mEq/L)	Sex			Odds ratio	95% CI
	Male	Female	Total		
<30	1,821	1,415	3,236	**1.415**	**1.266–1.582**
≥30 to <60	633	691	1,324	**0.76**	**0.67–0.86**
≥60	332	370	704	**0.77**	**0.657–0.903**

**Chloride levels**	**Indication––respiratory disease**			**Odds ratio**	**95% CI**
	**Yes**	**No**	**Total**		

<30	2,159	491	2,650	**2.71**	**2.271–3.235**
≥30 to <60	343	130	473	**0.749**	**0.601–0.933**
≥60	121	156	277	**0.193**	**0.15–0.248**

**Chloride levels**	**Indication––digestive disease**			**Odds ratio**	**95% CI**
	**Yes**	**No**	**Total**		

<30	278	2,372	2,650	**0.506**	**0.406–0.632**
≥30 to <60	63	410	473	1.11	0.832–1.479
≥60	78	199	277	**3.198**	**2.405–4.252**

*Statistical analysis was made by chi-square test; odds ratio was calculated considering values set by Taylor series. For all analyses, P-value was <0.001. All positive values are represented in bold type. α = 0.05*.

*95% CI, confidence interval of 95%; mEq/L, milliequivalents per liter*.

**Figure 1:**
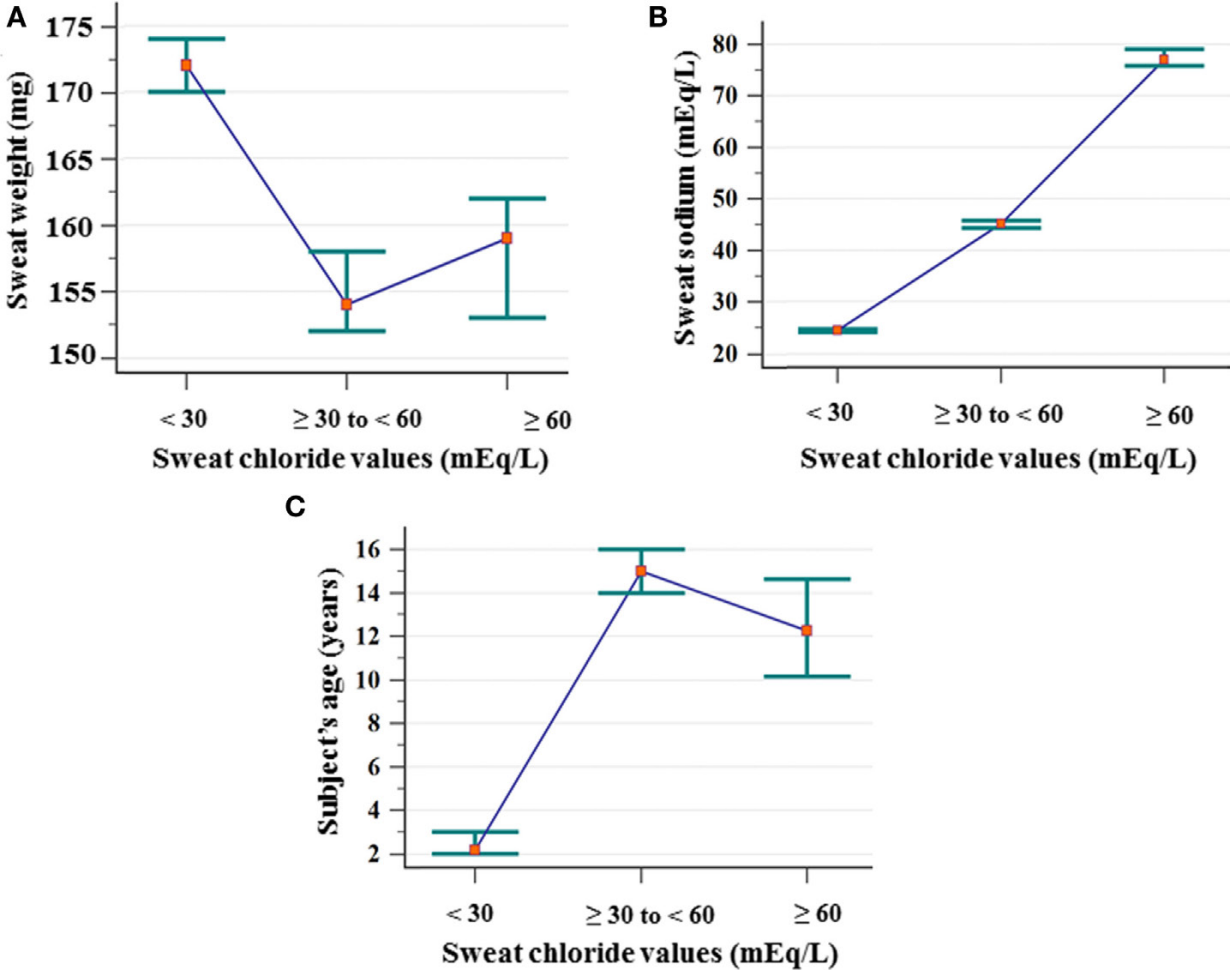
Association between the ST results considering chloride cutoff values and sweat weight (mg), sodium value (mEq/L), and subject’s age (years). **(A)** Sweat weight: (chloride <30 mEq/L) *N* = 3,249; mean of 175 ± 43; median of 172 (amplitude from 75 to 367); (chloride ≥30 to <60 mEq/L) *N* = 1,326; mean of 162 ± 41; median of 154 (amplitude from 78 to 299); (chloride ≥60 mEq/L) *N* = 702; mean of 164 ± 42; median of 159 (amplitude from 78 to 326). Chloride <30 mEq/L ≠ chloride ≥30 to <60 mEq/L, and chloride ≥60 mEq/L. **(B)** Sweat–sodium concentration: (chloride <30 mEq/L) *N* = 3,249; mean of 25.24 ± 7.03; median of 24.4 (amplitude from 10 to 68.5); (chloride ≥30 to <60 mEq/L) *N* = 1,326; mean of 45.82 ± 9.89; median of 45.11 (amplitude from 20.3 to 81.8); (chloride ≥60 mEq/L) *N* = 702; mean of 80.54 ± 17.98; median of 77 (amplitude from 42.9 to 149.7). All groups differ from each other. **(C)** Subject’s age: (<30 mEq/L) *N* = 3.233; mean of 6.66 ± 11.91; median of 2.17 (amplitude from 0 to 76.33); (chloride ≥30 to <60 mEq/L) *N* = 1,282; mean of 24.34 ± 21.74; median of 15 (amplitude from 0 to 85.58); (chloride ≥60 mEq/L) *N* = 621; mean of 21.27 ± 22.52; median of 12.25 (amplitude from 0 to 81.17). All groups differ from each other. Statistical analysis was performed by Kruskal–Wallis test. *P*-value for all analyses was <0.001. α = 0.05. The median is set in red marker and the 95% confidence interval is set in green line. mEq/L, milliequivalents per liter; ST, sweat test.

Figure 1 shows the association of ST results with the weight of sweat, sweat sodium value and subject’s age.

Table 2 presents the subjects’ age distribution at the time of the ST, as well as the comparison between ST results and reason for the examination indication. There was a higher prevalence of sweat samples with sweat chloride levels <30 mEq/L in subjects aged ≥6 months to <18 years. The CF diagnosis (chloride ≥60 mEq/L) showed higher prevalence in the group aged over 18 years. Higher prevalence ratio for the group older than 18 years of age with borderline sweat chloride values was also observed. The indication for the ST due to respiratory manifestations was more often in the group aged ≥6 months to <18 years. For digestive manifestation, indication was more often in the group under six months of age.

**Table 2:** Association between sweat chloride levels, sex of the subjects, and reason for indication of sweat test with age and sweat test result.

Chloride levels (mEq/L)	Age				Odds ratio	95% CI	Odds ratio	95% CI	Odds ratio	95% CI
	0 to <6 m	≥6 m to <18 years	≥18 years	Total
<30	426	2,579	230	3,235	1.959	1.605–2.391	3.947	3.49–4.465	0.102	0.087–0.12
≥30 to <60	46	657	587	1,290	**0.24**	**0.177–0.327**	**0.36**	**0.316–0.41**	**5.891**	**5.096–6.811**
≥60	95	322	255	674	**1.408**	**1.111–1.785**	**0.366**	**0.311–0.432**	**2.775**	**2.334–3.3**

**Indication by two factors^b^**	**0 to <6 m**	**≥6 m to <18 years**	**≥18 years**	**Total**	**Odds ratio**	**95% CI**	**Odds ratio**	**95 %CI**	**Odds ratio**	**95% CI**

No factor	167	405	5	577	**2.884**	**2.333–3.565**	**0.334**	**0.27–0.412**	**24.64**	**2.874–211.3^a^**
One factor	318	2,282	1	2,601	**0.421**	**0.345-0.513**	**2.449**	**2.009–2.984**	**0.061**	**0.007–0.522^a^**
Two factors	31	188	0	219	0.916	0.619–1.355	1.108	0.749–1.64	–	–

**Sex**	**0 to <6 m**	**≥6 m to <18 years**	**≥18 years**	**Total**	**Odds ratio**	**95% CI**	**Odds ratio**	**95 %CI**	**Odds ratio**	**95% CI**

Male	312	1,996	439	2,747	1.157	0.969–1.381	**1.497**	**1.331–1.684**	**0.539**	**0.471–0.618**
Female	243	1,559	634	2,436	1	–	1	–	1	–

**Respiratory**	**0 to <6 m**	**≥6 m to <18 years**	**≥18 years**	**Total**	**Odds ratio**	**95% CI**	**Odds ratio**	**95% CI**	**Odds ratio**	**95% CI**

Yes	282	2,337	1	2,620	**0.28**	**0.23–0.341**	**3.668**	**3.015–4.463**	**0.059**	**0.007–0.505**
No	234	538	5	777	1	–	1	–	1	–

**Digestive**	**0 to <6 m**	**≥6 m to <18 years**	**≥18 years**	**Total**	**Odds ratio**	**95% CI**	**Odds ratio**	**95% CI**	**Odds ratio**	**95% CI**

Yes	98	321	0	419	**1.87**	**1.458–2.398**	**0.544**	**0.424–0.697**	–	–
No	418	2,554	6	2,978	1	–	1	–	–	–

**Indication by two factors^b^**	**Sweat test-chloride values (mEq/L)**				**Odds ratio**	**95% CI**	**Odds ratio**	**95% CI**	**Odds ratio**	**95% CI**
	**<30**	**≥30 to <60**	**≥60**	**Total**						

No one	368	99	110	577	**0.417**	**0.344–0.507**	**1.356**	**1.064–1.728**	**3.746**	**2.889–4.858**
One factor	2,127	342	135	2,604	**2.328**	**1.95–2.778**	**0.768**	**0.617–0.956**	**0.252**	**0.196–0.324**
Two factors	155	32	32	219	**0.666**	**0.492–0.902**	1.063	0.721–1.567	**2.051**	**1.379–3.05**

*^b^No factor = absence of respiratory and digestive symptoms at the moment that the ST was performed; one factor = presence of respiratory or digestive symptoms at the moment that the ST was performed; two factors = presence of respiratory and digestive symptoms at the moment that the ST was performed. Statistical analysis was made by chi-square test; odds ratio was calculated considering values set by Taylor series, except for the data marked with ^a^ (odds ratio was calculated considering values set by Fisher’s exact test)*.

*For all analyses, P-value was <0.001. All positive values are represented in bold type. α = 0.05. 95% CI, confidence interval of 95%; m, months; mEq/L, milliequivalents per liter; ST, sweat test*.

There was no association between the reason for ST indication and the sex of the subjects [respiratory symptoms (*p* = 0.362), digestive symptoms (*p* = 1), or nutritional symptoms (*p* = 0.384)]. However, male patients were less prevalent in the group aged over 18 years and showed higher volume of sweat and lower levels of sweat sodium, sweat chloride, and chloride/sodium ratio (Tables 2 and 3).

Subjects referred to the ST because of respiratory symptoms were older and presented higher sweat weight, lower levels of sweat chloride, sweat sodium and chloride/sodium ratio, as well as higher sodium–chloride level. For the indication due to digestive reasons, there was no association with sweat weight (*p* = 0.426) (Table 3). However, there was association with digestive symptom and higher levels of sweat chloride, sodium and chloride/sodium ratio, as well as subject’s younger age and lower sodium–chloride level (Table 3).

**Table 3:** Association between sweat weight, sex of the subjects, sweat chloride, sweat sodium, and reason for indication of sweat test.

Variable	Category	N	Mean	Standard variation	Median	Minimum	Maximum
**Sweat weight (mg)**							
Sex	Male	2,786	174	43	172	76	367
	Female	2,476	165	41	161	75	337
Respiratory	Yes	2,623	170	41	168	76	367
	No	777	159	38	157	75	295
Indication by two factors^a,b^	No factor	577	157	37	155	75	276
	One factor	2,604	170	41	167	76	367
	Two factors	219	171	47	164	76	296

**Age (years)**							
Sex	Male	2,712	10.41	15.75	4	0	80.83
	Female	2,409	15.65	20.44	6	0	85.58
Respiratory	Yes	2,620	4.28	4.03	3	0	44
	No	777	4.2	4.94	2	0	19
Digestive	Yes	419	3.42	4.27	2	0	17
	No	2,978	4.38	4.24	3	0	44

**Sweat sodium (mEq/L)**							
Sex	Male	2,786	36.16	20.77	29.3	10	149.7
	Female	2,476	39.66	21.8	32.75	10.1	145.08
Respiratory	Yes	2,623	30.52	15.14	26.4	11.1	119.3
	No	777	41.79	25.01	32.2	10	149.7
Digestive	Yes	419	39.26	25.23	29.6	10	124.6
	No	2,981	32.23	17.17	27.2	11.1	149.7
Indication by two factors^c^	No one	577	41.38	23.83	32.2	12.9	149.7
	One factor	2,604	31.03	16.17	26.5	10	124.6
	Two factors	219	35.86	21.7	28	11.6	117

**Sweat chloride (mEq/L)**							
Sex	Male	2,786	32.27	26.44	22.25	10	159.2
	Female	2,476	36.07	27.86	25.8	10	158.6
Respiratory	Yes	2,623	23.91	18.6	18.3	10	136.2
	No	777	38.19	33.29	22.6	10	152.3
Digestive	Yes	419	36.94	33.93	21	10	141.8
	No	2,981	25.8	21.38	18.8	10	153.3
Indication by two factors^c^	No factor	577	37.25	32	22.6	10	152.3
	One factor	2,604	24.42	19.66	18.4	10	141.8
	Two factors	219	33.33	30.82	19.7	10.4	130.4

**Chloride/sodium**							
Sex	Male	2,786	0.835	0.229	0.806	0.31	2.1
	Female	2,476	0.854	0.239	0.834	0.29	2.31
Respiratory	Yes	2,623	0.747	0.182	0.731	0.31	1.96
	No	777	0.824	0.26	0.776	0.32	2.16
Digestive	Yes	419	0.843	0.26	0.791	0.42	2.16
	No	2,981	0.754	0.193	0.733	0.31	1.96
Indication by two factors^d^	No factor	577	0.81	0.26	0.77	0.32	1.81
	One factor	2,604	0.75	0.18	0.73	0.31	2.16
	Two factors	219	0.83	0.26	0.78	0.42	1.81

**Sodium–chloride**							
Respiratory	Yes	2,623	6.608	7.752	6.9	−66.6	33.7
	No	777	3.602	12.807	6.2	−60.7	42.4
Digestive	Yes	419	2.32	12.382	5.4	−51.4	26.6
	No	2,981	6.427	8.592	7.04	−66.6	42.4
Indication by two factors^e^	No factor	577	4.13	12.96	6.8	−60.7	42.4
	One factor	2,604	6.6	7.69	6.9	−66.6	33.7
	Two factors	219	2.53	12.51	5.6	−49.6	22

*^a^No factor = absence of respiratory and digestive symptoms at the moment that the ST was performed; one factor = presence of respiratory or digestive symptoms at the moment that the ST was performed; two factors = presence of respiratory and digestive symptoms at the moment that the ST was performed*.

*^b^The no factor group showed difference regarding the others groups*.

*^c^All the groups are different among them*.

*^d^One factor group showed difference regarding the others groups*.

*^e^Two factor group showed difference regarding the others groups. Statistical analysis was made by Mann–Whitney test and Kruskal-Wallis test*.

For all analyses, P-value was <0.001, except* p = 0.001. α = 0.05.

*N, number of subjects; mg, milligram; mEq/L, milliequivalents per liter; ST, sweat test*.

**Figure 2:**
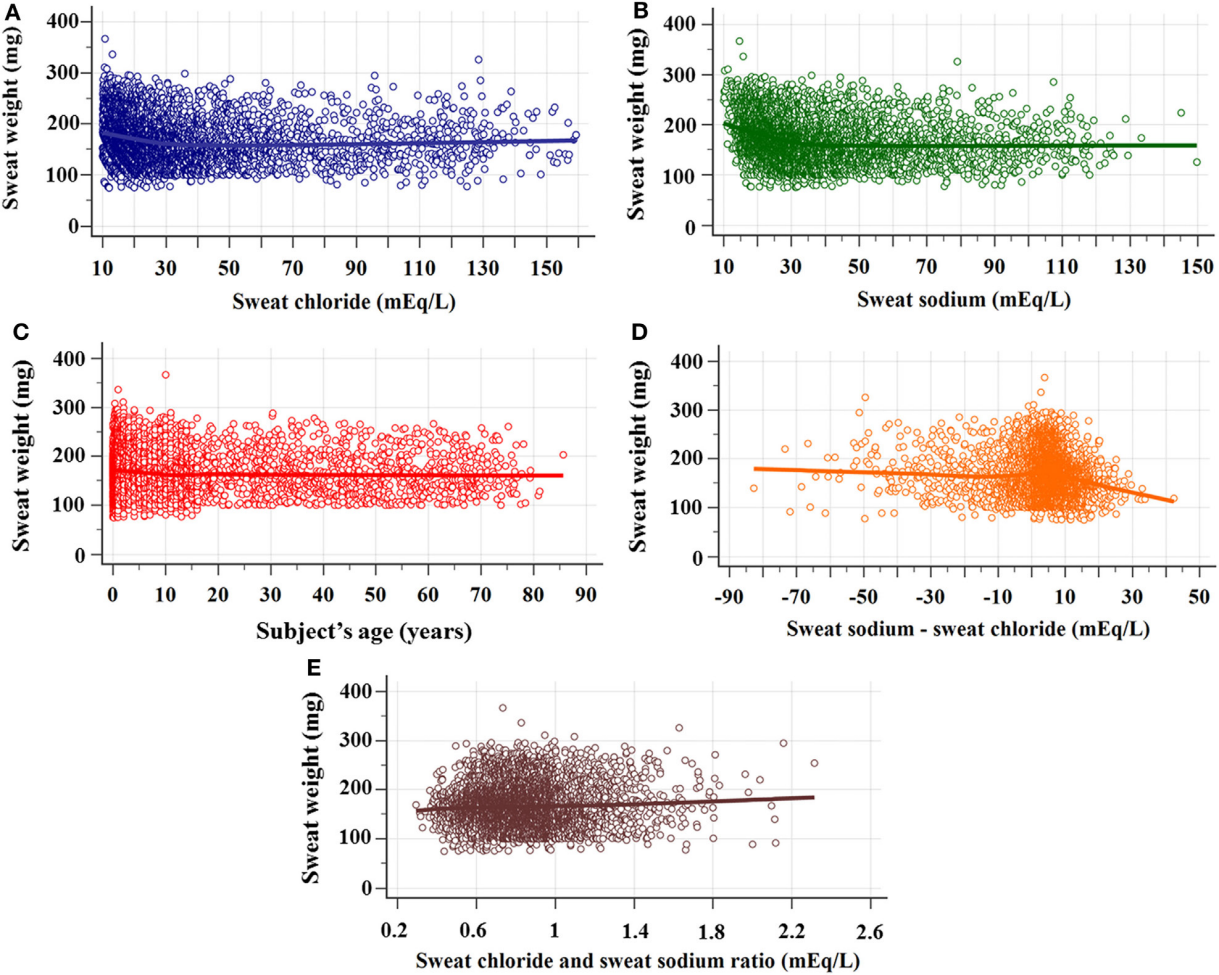
Correlation between sweat weight and sweat chloride and sodium concentrations, subject’s age, difference between sweat chloride and sodium concentrations, and sodium/chloride ratio. **(A)** Correlation between sweat weight and chloride concentrations. *N* = 5,277; rho = −0.201 (95% CI = −0.227 to −0.175), *p* < 0.0001. **(B)** Correlation between sweat weight and sweat sodium concentrations. *N* = 5,277; rho = −0.257 (95% CI = −0.282 to −0.232), *p* < 0.001. **(C)** Correlation between sweat weight and subject’s age. *N* = 5,136; rho = –0.075 (95% CI = –0.102 to –0.048), *p* < 0.0001. **(D)** Correlation between sweat weight and the difference between sweat sodium and chloride concentrations. *N* = 5,277; rho = –0.094 (95% CI = –0.121 to 0.067), *p* < 0.0001. **(E)** Correlation between sweat weight and chloride/sodium ratio. *N* = 5,277; rho = 0.007 (95% CI = –0.02 to 0.034), *p* = 0.621. The statistical analysis was performed by Spearman’s rank correlation test. α = 0.05. 95% CI, confidence interval of 95%.

Figure 2 brings the correlation between sweat weight and levels of sweat chloride and sweat sodium, subject’s age, difference between sodium and chloride concentrations and chloride/sodium ratio.

**Figure 3:**
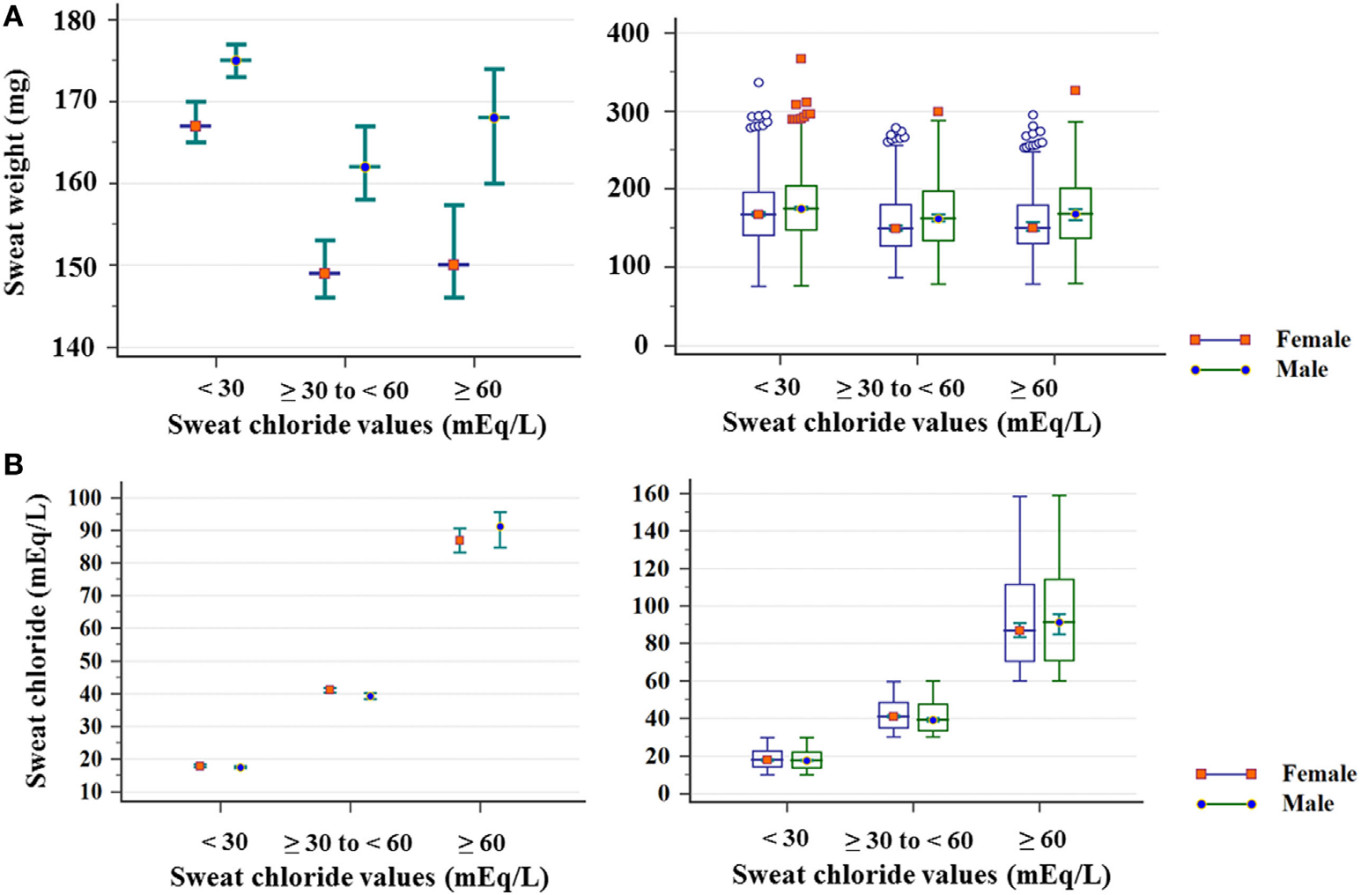
Distribution of sex of subject, considering sweat chloride (mEq/L) and sweat weight. **(A)** Sweat weight (mg). (chloride <30 mEq/L) Female: *N* = 1,415; mean of 171 ± 43; median of 167 (amplitude from 75 to 337); male: *N* = 1,821; mean of 178 ± 43; median of 175 (amplitude from 76 to 367) (*p* < 0.001); (chloride ≥30 to <60 mEq/L) female: *N* = 691; mean of 156 ± 38; median of 149 (amplitude from 87 to 279); male: *N* = 633; mean of 168 ± 44; median of 162 (amplitude from 78 a 299) (*p* < 0.001); (chloride ≥60 mEq/L) Female: *N* = 370; mean of 157 ± 39; median of 150 (amplitude from 78 to 295); male: *N* = 332; mean of 170 ± 44; median of 168 (amplitude from 79 to 326) (*p* < 0.001). **(B)** Sweat chloride concentrations obtained in the sweat test. (<30 mEq/L) Female: *N* = 1,821; mean of 18.15 ± 5.26; median of 17.4 (amplitude from 10 to 29.9); male: *N* = 1,415; mean of 18.54 ± 5.29; median of 17.7 (amplitude from 10 to 29.9) (*p* = 0.038); (chloride ≥30 to < 60 mEq/L) female: *N* = 691; mean of 42.08 ± 8.29; median of 41.1 (amplitude from 30 to 59.8); male: *N* = 633; mean of 40.89 ± 8.28; median of 39.3 (amplitude from 30 to 59.9) (*p* = 0.006); (chloride ≥60 mEq/L) female: *N* = 370; mean of 91.87 ± 24.53; median of 86.85 (amplitude from 60 to 158.6); male: *N* = 332; mean of 93.28 ± 25.06; median of 91.2 (amplitude from 60 to 159.2) (*p* = 0.618). The statistical analysis was performed by Mann–Whitney test. α = 0.05. The median is set in red marker and the 95% confidence interval is set in green line. The second graphic is a box plot. mEq/L, milliequivalents per liter.

Figure 3 shows the sex of the subjects distribution by the result of CF diagnosis regarding the sweat weight and the sweat chloride.

**Figure 4:**
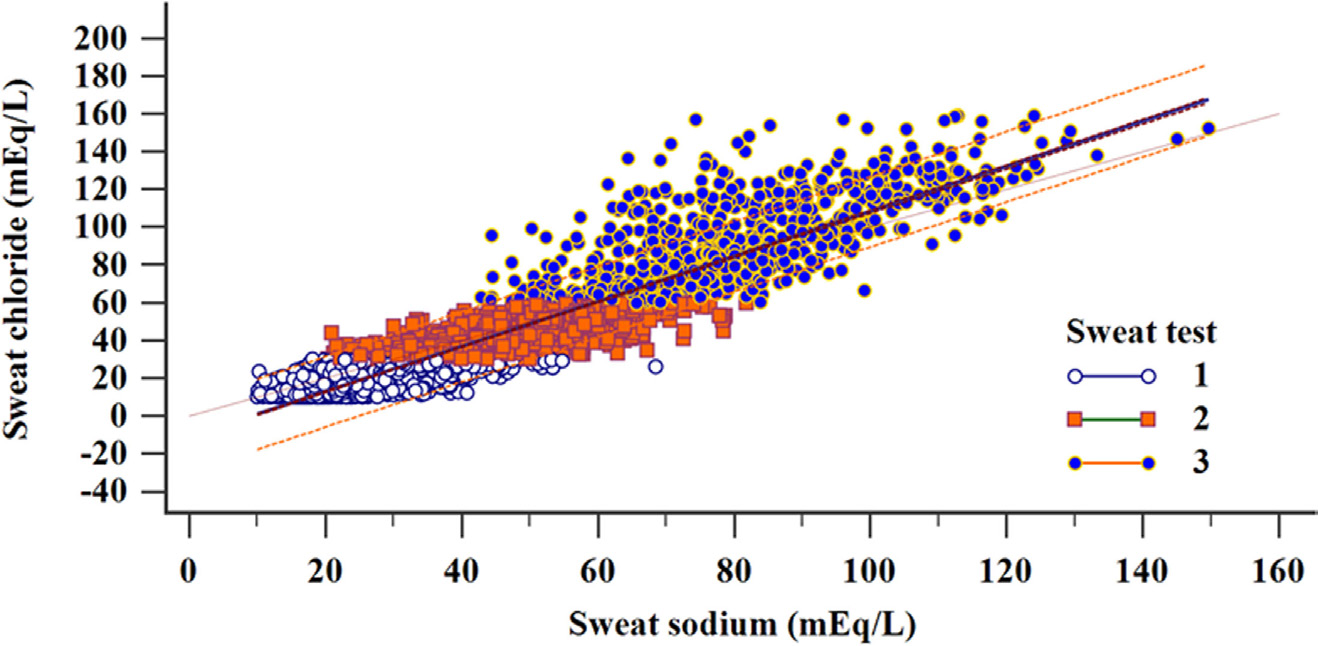
Regression and correlation of sweat sodium and chloride concentrations distributed according to the diagnostic results considering the sweat test values (total sample). Linear regression: *N* = 5,277; *R*^2^ = 0.877; *y* = −11.05 + 1.1932*x* (*p* < 0.001). Correlation: *N* = 5,277; correlation coefficient *r* = 0.9365; 95% CI = 0.9331–0.9397 (*p* < 0.001); (1) *N* = 3,249; *R*^2^ = 0.4347; *y* = 5.8091 + 0.4955*x* (*p* < 0.001); (2) *N* = 1,324; *R*^2^ = 0.4913; *y* = 14.5228 + 0.5888*x* (*p* < 0.001); (3) *N* = 702; *R*^2^ = 0.5336; *y* = 11.4797 + 1.0064*x* (*p* < 0.001). (i) chloride <30 mEq/L; (ii) chloride ≥30 to <60 mEq/L; and (iii) chloride ≥60 mEq/L. α = 0.05. mEq/L, milliequivalents per liter; 95% CI, confidence interval of 95%.

Figure 4 shows the correlation between the levels of sweat sodium and sweat chloride by the result of CF diagnosis.

In addition, the association between the IRT inclusion in our sample and sex of the subjects, CF diagnosis, and age are presented in Table 4. The association with indication to the ST performance was not made regarding the bias by the IRT inclusion.

**Table 4:** Association between immunoreactive trypsinogen (IRT) inclusion in our center and subject’s sex and age and cystic fibrosis diagnosis.

Chloride levels (mEq/L)	Inclusion of IRT			Odds ratio	95% CI
	After	Before	Total		
<30	503	2,749	3,252	**0.313**	**0.275–0.357**
≥30 to <60	500	824	1,324	**2.603**	**2.27–2.985**
≥60	242	447	689	**1.929**	**1.625–2.289**

**Sex**	**Yes**	**No**	**Total**	**Odds ratio**	**95% CI**

Male	574	2,202	2,776	**0.702**	**0.618–0.797**
Female	670	1,804	2,474	1	–

**Subject’s age**	**Yes**	**No**	**Total**	**Odds ratio**	**95% CI**

0 to <6 months	52	515	567	**0.3**	**0.224–0.403**
≥6 months to <18 years	633	2,916	3,549	**0.395**	**0.345–0.451**
≥18 years	529	541	1,070	**4.898**	**4.236–5.663**

*Statistical analysis was made by chi-square test; odds ratio was calculated considering values set by Taylor series. For all analyses, P-value was <0.001. All positive values are represented in bold type. α = 0.05*.

*95% CI, confidence interval of 95%; IRT, immunoreactive trypsinogen; mEq/L, milliequivalents per liter*.

In our sample, 408 subjects were screened for at least F508del mutation in the *CFTR* gene. From these subjects, we found (i) 169/408 (41.4%) patients with unknown mutation in the *CFTR* gene, or with one or two mutations in the *CFTR* gene belonging to classes IV, V, or VI; (ii) 87/408 (21.3%) patients with one mutation in the *CFTR* gene belonging to class I, II, or III, and unknown mutation or one mutation in the *CFTR* gene belonging to class IV, V, or VI; (iii) 152/408 (37.3%) patients with two identified mutations in the *CFTR* gene belonging to class I, II, and/or III (see Table 5).

**Table 5:** Distribution of patients for the *CFTR* gene genotype and classes of identified mutations*.

Genotype	N	Group of patients with cystic fibrosis
Unknown/unknown	137	169/408 (41.4%) patients with unknown mutation in the *CFTR* gene, or with one or two mutations in the *CFTR* gene belonging to classes IV, V, or VI
V562I/unknown	1	
G576A/R668C	3	
p.Glu528G > A/TG11-5T	3	
R334W/R334W	1	
D110H/V232H	1	
I507V/unknown	2	
D614G/unknown	4	
F508del/unknown	61	87/408 (21.3%) patients with mutation in the *CFTR* gene belonging to class I, II, or III, and unknown mutation or one mutation in the *CFTR* gene belonging to class IV, V, or VI
G542X/unknown	6	
G542X/P205S	2	
G542X/R334W	2	
622-2 A > G/711 + 1G > T	1	
G542X/I618T	3	
3120+ 1G > A/L206W	3	
F508del/D1152H	2	
F508del/R334W	2	
R1066C/R334W	1	
F508del/P205S	3	
R1162X/unknown	1	
F508del/F508del	88	152/408 (37.3%) patients with two mutations identified in the *CFTR* gene belonging to class I, II, and/or III
F508del/G542X	22	
F508del/N1303K	8	
F508del/R1162X	8	
F508del/R553X	5	
F508del/1584-18672pbA > G	4	
F508del/c.1717 − 1G > A	3	
3120 + 1G > A/R1066C	3	
F508del/2183AA > G	1	
F508del/2184insA	1	
F508del/6B to 16 exon duplication	2	
F508del/G85E	2	
F508del/S549R (T > G)	2	
G542X/2183AA > G	1	
G542X/R1162X	2	
F508del/S4X	3	
F508del/R1066C	4	
F508del/1812 − 1G > A	4	
R1162X/R1162X	4	
2183AA > G/2183AA > G	2	
3120 + 1G > A/3120 + 1G > A	1	

*F508del/other mutation = 136/408 (33.3%); F508del/F508del = 88/408 (21.6%)*.

*CFTR, cystic fibrosis transmembrane regulator; N, sample size*.

The *CFTR* genotype was associated with sex, reason for indication of ST, result achieved from ST, age of subjects, and ST markers regarding *CFTR* mutation screening genotype and F508del genotype (Tables 6 and 7).

**Table 6:** Association between sex of the subject, reason for indication of sweat test, result achieved from sweat test, and subjects’ ages with *CFTR* mutation screening.

Chloride levels (mEq/L)	*CFTR* genotype				Odds ratio	95% CI	Odds ratio	95% CI	Odds ratio	95% CI
	IM/IM	IM/NIM	NIM/NIM	Total						
<30	6^a^	0	6	12	1.429	0.453–4.51	**–**	**–**	1.712	0.542–5.407
≥30 to <60	4^b^	3	48	55	**0.089**	**0.023–0.252**^a^	**0.185**	**0.056–0.607**^a^	**16.42**	**7.193–37.47**
≥60	159	84	98	341	**4.98**	**2.461–10.08**	**6.973**	**2.135–22.77**	**0.097**	**0.05–0.186**

**Subject’s age**										

0 to <6 m	50	17	1	68	**5.242**	**2.922–9.401**	1.294	0.703–2.383	**0.018**	**0.001–0.095**^a^
≥6 m to <18 years	106	47	75	228	**1.664**	**1.106–2.504**	0.916	0.565–1.483	**0.634**	**0.421–0.954**
≥18 years	9	21	74	104	0.085	0.041–0.175	0.732	0.425–1.263	**7.14**	**4.34–11.75**

**Respiratory**										

Yes	36	17	18	71	**0.356**	**0.188–0.674**	1.425	0.682–2.979	**4.118**	**1.678–10.1**
No	78	19	8	105	1	–	1	–	1	–

**Chloride levels (mEq/L)**	**Presence of F508del mutation**				**Odds ratio**	**95% CI**	**Odds ratio**	**95% CI**	**Odds ratio**	**95% CI**
	**+/+**	**+**/**−**	**−/−**	**Total**						

<30	2	4	6	12	0.722	0.075–3.479^a^	1	0.216–3.815^a^	1.225	0.388–3.863
≥30 to <60	2	4	49	55	**0.117**	**0.014–0.463^a^**	**0.131**	**0.046–0.372^a^**	**13.19**	**5.501–31.62**
≥60	84	128	129	341	**5.148**	**1.819–14.57**	**4.432**	**2.052–9.574**	**0.133**	**0.069–0.257**

**Subject’s age**										

0 to <6 m	31	29	8	68	**4.042**	**2.318–7.048**	1.701	0.997–2.902	**0.121**	**0.056–0.261**
≥6 m to <18 years	52	83	93	228	1.116	0.691–1.804	1.522	0.99–2.341	**0.642**	**0.431–0.957**
≥18 years	5	18	81	104	**0.13**	**0.051–0.33**	0.344	0.197–0.602	**6.799**	**4.036–11.46**

**Sex**										

Male	53	60	69	182	**2.242**	**1.385–3.63**	0.971	0.641–1.469	**0.589**	**0.396–0.877**
Female	35	76	115	226	1	–	1	–	1	–

**Respiratory**										

Yes	12	32	27	71	**0.282**	**0.136–0.586**	1.094	0.597–2.007	**3.413**	**1.668–6.985**
No	44	45	16	105	1	–	1	–	1	–

*NIM––unknown mutation in the *CFTR* gene, or with one or two mutations in the *CFTR* gene belonging to classes IV, V, or VI; IM––known mutations in the *CFTR* gene belonging to class I, II, and/or III; (+) shows that the F508del allele is present; (−) shows that the F508del allele is not present*.

*^a^The cystic fibrosis patients showed the following genotypes: four patients F508del/N1303K and two patients F508del/F508del*.

*^b^One patient F508del/S4X, one patient F508del/N1303K, and two patient F508del/F508del. Statistical analysis was made by chi-square test; odds ratio was calculated considering values set by Taylor series, except for the data marked with ^a^ (odds ratio was calculated considering values set by Fisher’s exact test)*.

*For all analyses, P-value was <0.002. All positive values are represented in bold type. α = 0.05*.

*CFTR, cystic fibrosis transmembrane regulator; 95% CI, confidence interval of 95%; IM, identified mutation; m, months; mEq/L, milliequivalents per liter; NIM, non-identified mutation*.

**Table 7:** Association between sweat test markers and *CFTR* mutation screening.

Variable	Category	N	Mean	Standard Variation	Median	Minimum	Maximum
***CFTR*** mutation							
Sweat weight (mg)^a^	IM/IM	169	163	4.4	161	79	326
	IM/NIM	87	174	4.6	174	78	276
	NIM/NIM	152	160	3.9	152	96	268
Sweat sodium (mEq/L)^a^	IM/IM	169	84.16	21.86	83.7	10.1	145.08
	IM/NIM	87	85.51	18.16	85.2	44.4	128.76
	NIM/NIM	152	68.62	20.76	67.2	14.23	113.8
Sweat chloride (mEq/L)^b^	IM/IM	169	107.29	27.08	109.42	13.1	159.2
	IM/NIM	87	107.03	23.41	113.6	46.8	152.5
	NIM/NIM	152	71.03	25.68	66	12.4	140.16
Sodium–chloride^b^	IM/IM	169	−23.13	18.21	−21.1	−82.7	22
	IM/NIM	87	−21.52	17.5	−19.2	−71.97	13.27
	NIM/NIM	152	−2.41	11.71	−0.46	−46.01	32.7
Chloride/sodium^b^	IM/IM	169	1.292	0.261	1.25	0.57	2.11
	IM/NIM	87	1.269	0.255	1.226	0.8	2.12
	NIM/NIM	152	1.029	0.174	1.007	0.49	1.73

**F508del mutation screening**							
Sweat sodium (mEq/L)^c^	+/+	88	84.4	20.8	84.95	10.1	124.6
	+/−	136	84.02	21.71	83.05	30.2	145.08
	−/−	184	71.95	21.15	70.35	14.23	118.81
Sweat chloride (mEq/L)^c^	+/+	88	107.69	25.86	106.8	13.1	159.2
	+/−	136	105.52	26.89	111.36	21.6	158.6
	−/−	184	78.33	29.51	72.61	12.4	152.5
Sodium–chloride^c^	+/+	88	−23.27	16.78	−23.2	−64.3	11.5
	+/−	136	−21.5	18.39	−18.65	−82.7	22
	−/−	184	−6.39	15.88	−1.85	−73.42	32.7
Chloride/sodium^c^	+/+	88	1.294	0.23	1.263	0.87	1.98
	+/−	136	1.273	0.275	1.21	0.57	2.12
	−/−	184	1.077	0.22	1.03	0.49	2.04

*NIM––unknown mutation in the *CFTR* gene, or with one or two mutations in the *CFTR* gene belonging to classes IV, V, or VI; IM––known mutations in the *CFTR* gene belonging to class I, II, and/or III; (+) shows that the F508del allele is present; (−) shows that the F508del allele is not present*.

*^a^IM/NIM genotype showed difference regarding the other groups*.

*^b^NIM/NIM genotype showed difference regarding the other groups*.

*^c^−/− genotype showed difference regarding the other groups*.

*Statistical analysis was made by Kruskal–Wallis test. For all analyses, P-value was <0.001. α = 0.05*.

*CFTR, cystic fibrosis transmembrane regulator; IM, identified mutation; mEq/L, milliequivalents per liter; mg, milligram; N, number of subjects; NIM, non-identified mutation*.

**Figure 5:**
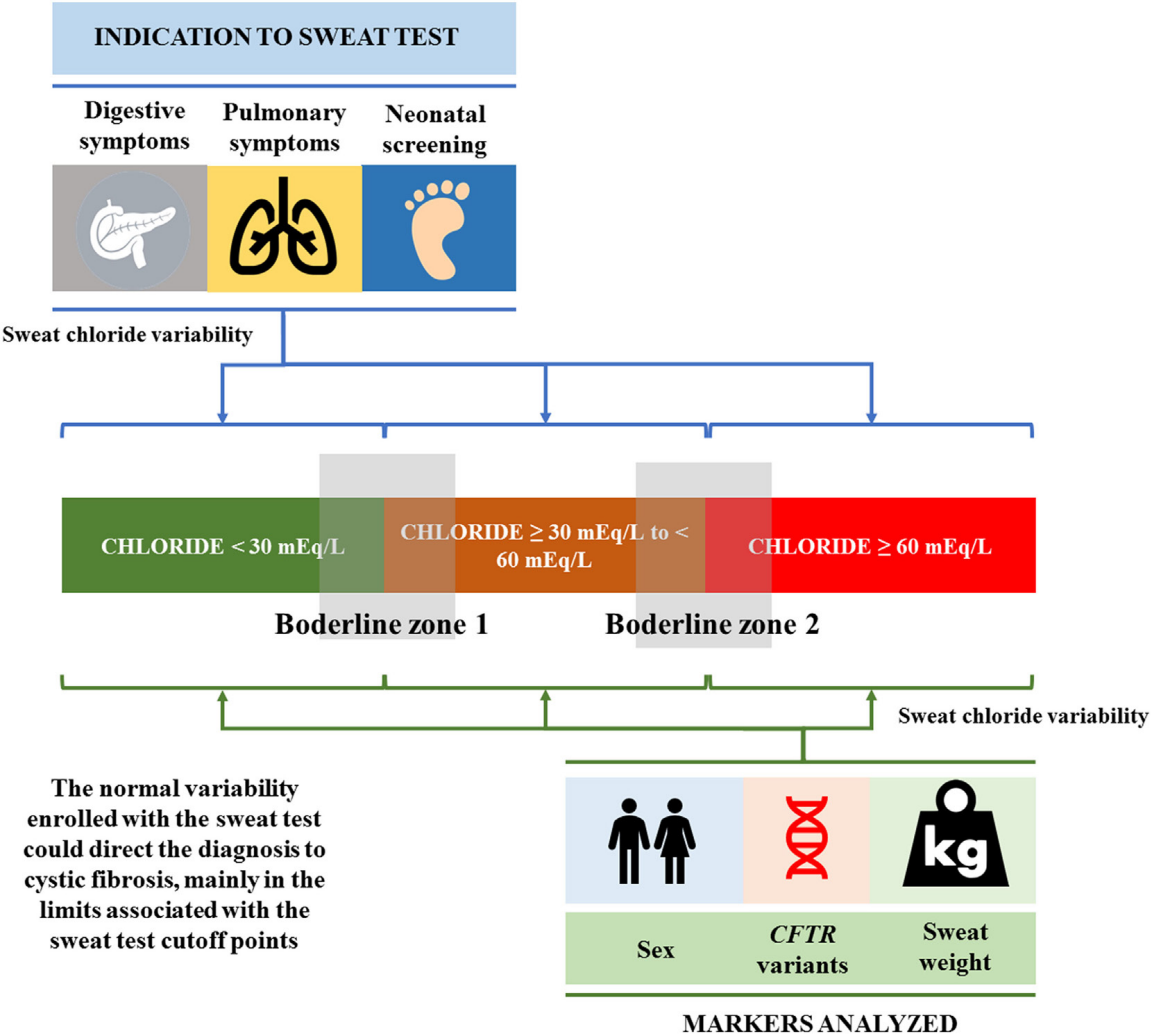
The normal variability enrolled with the sweat test, which could direct the diagnosis to cystic fibrosis, mainly in the limits (borderline zones 1 and 2) associated with the sweat test cutoff points. In addition, sex, *CFTR* mutation, sweat weight, digestive symptoms, pulmonary symptoms, and neonatal screening influenced the values achieved in the sweat test, and this influence is present in all possible sweat test classes: (i) <30 mEq/L; (ii) ≥30 to <60 mEq/L; and (iii) ≥60 mEq/L. *CFTR*, cystic fibrosis transmembrane regulator.

The normal variability enrolled with the ST could direct the diagnosis to CF, mainly in the limits associated with the ST cutoff points. In addition, the sex, *CFTR* mutation, sweat weight, digestive symptoms, pulmonary symptoms, and neonatal screening influenced the values achieved in the ST, and this influence is present in all possible ST classes: (i) <30 mEq/L; (ii) ≥30 to <60 mEq/L; and (iii) ≥60 mEq/L (Figure 5).

## Discussion

High levels of sweat chloride with the presence of CF indicative signs and symptoms have been the gold standard for the CF diagnosis for more than 70 years (3). The amounts of ions in sweat have high specificity and sensitivity to demonstrate dysfunction or absence of chloride channels (CFTR protein) (18). However, despite the efficacy and importance of the ST for CF diagnosis, many methodological aspects of the stages related to its realization and interpretation continue and should be discussed as follows: (i) How do sweat chloride and sweat sodium concentrations vary with age? (15, 19); (ii) Is it necessary to measure sweat chloride alone or together with the analysis of sweat sodium? (15, 20); (iii) Which are the most appropriate types of waves and current intensity to stimulate sweating in the ST? (4); (iv) How are the ST stages performed at different reference centers? (21); How has the evolution of the ST been since its implementation for nearly 70 years? (3); Are the values of electrolytes obtained in the ST dependent on the variants of the *CFTR* gene? (16).

The importance of the ST is increasing day by day, and recently the sweat chloride level has been described as a predictive marker of CF severity lung disease (22).

In this context, in the present study we intended to demonstrate the characterization of the data presented in the ST regarding the influence of subject’s sex and age, *CFTR* mutation screening, and, mainly, ST clinical indication (nutritional, respiratory, and digestive symptoms). All aspects analyzed were discussed in part as follows.

### ST Clinical Indication

Our laboratory, at a university referral center, receives requests to measure sweat sodium and sweat chloride ions in sweat from several specialties, mainly pediatric care. STs were more frequently requested for patients with chronic respiratory and digestive diseases.

Most patients with non-classic phenotype show symptoms later in life, often during adolescence or later, namely, less severe form of the disease, pancreatic sufficiency, mild chronic respiratory symptoms, and obstruction of the vas deferens with azoospermia. Levels of sweat chloride may be normal, abnormal, or borderline in patients with non-classical phenotypes. However, these levels are lower in patients with classic phenotypes (16, 23). In this context, the clinical manifestation could drive to the early or late CF diagnosis by the ST. In addition, early CF diagnosis provides numerous benefits, such as prevention of early malnutrition, reduced pulmonary complications, and long-term minor deterioration in lung function, allowing higher life quality and expectancy.

Our study found that the main reason for the ST requests was related to respiratory signs and symptoms (77.1%), followed by digestive symptoms (12.3%) and nutritional symptoms (11.5%). These data are in line with a recent study performed in France with 523 STs (24). Moreover, the ST request due to digestive manifestations prevailed in the group aged under six months, showing higher correlation with chloride values ≥60 mEq/L.

According to Ribeiro et al. (25), exocrine pancreatic insufficiency is often the earliest and most prevalent symptom in about 75% of patients with CF at birth, in 80–85% at the end of the first year and in 90% at adult age (25). As digestive symptoms are early identified, it allows differential diagnosis (26, 27). This fact was observed in our data, where the indication of ST due to digestive symptoms was also associated with higher values of sweat chloride, sweat sodium, and chloride/sodium ratio, as well as lower sodium–chloride level.

The low concordance between the ST indication by respiratory symptoms and the CF diagnosis might be related with the wide variability in the pulmonary symptoms, in addition to the higher number of diseases associated with pulmonary tract as compared with digestive tract. In this context, many solicitations of ST were performed to exclude the CF diagnosis hypothesis among many other pulmonary tract diseases.

Finally, the mutual association between respiratory and digestive symptoms enabled a better odds ratio for CF diagnosis and a lower odds ratio to achieve normal values in the ST. In literature, the clinical decision rule might be cited as a reliable index of clinical suspicion and timely referral for sweat testing in settings without newborn screening (NBS) programs, and may also be applied to false-negative individuals where such programs already exist (28).

### Demographic Data Related with the Variability Presented in the ST

In this study, the analysis of a larger ST sample size indicated that the levels of sweat chloride and sweat sodium vary with age and sex.

#### Sex

In this study, males showed higher sweat weight. In addition, males showed higher sweat chloride levels only among those with sweat chloride values <30 mEq/mL cutoff in the ST as compared with females. This is likely to be explained by the fact that the sweat volume was higher in men due to the production of their sweat glands. Males have fewer active glands with higher sweat rate per gland and increased response to cholinergic and beta-adrenergic stimulation, as compared with females (5). In this context, these observations indicate prospects for the diagnosis of functional changes of the CFTR protein that include the difference between sexes (29).

#### Subject’s Age

Notably, the onset age of CF symptoms is widely variable: they may occur in the first years of life, childhood, adolescence, or even adulthood. This complex onset of symptoms can be explained by environmental factor, modifier genes, and different mutations in the *CFTR* (1). Therefore, the CF diagnosis should be made as soon as possible, with a high degree of certainty due to medical, financial, and psychosocial implications. Moreover, in our study group, we showed the correlation between subject’s age and sweat chloride levels regarding the ST results (19).

Subjects under six months and over 18 years of age showed greater probability of positive ST (≥60 mEq/L) and higher prevalence of sweat samples with chloride levels <30 mEq/L. This can be explained by the fact that STs had been indicated for children younger than six months due to clear clinical signs and probability of a CF diagnosis.

The highest prevalence ratio for borderline sweat chloride levels could be observed in the group over 18 years of age. The CFTR dysfunction causes a series of disorders in the organism, and the ST does not always distinguish CF from other pathological conditions also mediated by CFTR (30). However, we observed high probability to perform the ST with values ≥60 mEq/L in patients over 18 years of age, and this fact can be associated with the exclusion of other diseases, mainly for lung pathologies, during the first years of life. After the exclusion of common diseases, as asthma, the indication for the ST with a positive result could show higher probability.

The indication of the ST due to respiratory symptoms prevailed among subjects aged six months to 18 years. Lung diseases are more frequent after six months of age and their presence and deterioration have been assessed by lung function tests, bacteriological markers, and high-resolution computed tomography (31). The early diagnosis should be done once the lungs of patients with CF are often colonized and infected with microorganisms that cause damage to the epithelial surface at early childhood. Chronic infections are the main reason for reduced lung function and are associated with increased morbidity and mortality in CF (32).

Finally, clinical indication should consider the subject’s age in order to induce the ST performance. Even if the NBS was performed, the presence of false-negative tests may occur. In the presence of IRT false-negative test and symptoms related with CF, the ST performance should be recommended.

### Correlation between Sweat Chloride and Sweat Sodium

The amounts of sweat sodium did not add discriminatory value to CF diagnosis. Current guidelines do not recommend using amounts of sweat sodium to CF diagnosis. However, some laboratories use this value for quality control purposes. The use of sweat sodium as quality control is given by the positive correlation between both ions (3), and this fact was showed in our study.

The association between sweat chloride and sweat sodium parameter to the ST quality control was analyzed by us previously, and we considered that the sweat sodium may be an indicative for quality discrimination, mainly in cases of doubts regarding the sweat chloride values (20). Even so, other studies should be done to clarify the biological variability, considering both ions dosage, observed in the ST among subjects that performed the test. Moreover, the studies should consider an ST performance overview, as a wide number of limitations and problems related in this examination at public and private centers is known (21). In this case, sweat sodium dosage should be stimulated in daily routine.

Finally, in all ages, levels of sweat sodium and sweat chloride were positively correlated, which had been documented in previous studies (3, 15).

### *CFTR* Mutation Influencing the Data Presented in the ST

In 1989, mutations in the *CFTR* gene were described as a causative factor of CF. The identification of the *CFTR* mutations directed the diagnosis toward molecular biology, allowing greater understanding of CF, revision of the diagnostic criteria, and new therapeutic possibilities (1, 33–35). Moreover, the *CFTR* mutations classification showed changes along the years (17). In this context, with the advances in the molecular field, more than 2,000 mutations have been identified in the *CFTR* gene (33); however, its association with the results presented in the ST is not well known. Advances in this field are important to define the reference and levels of sweat chloride in the ST for each class of mutations in the *CFTR* gene (16, 36).

In addition, variants in the *CFTR* gene may cause several clinical phenotypes, such as chronic sinusitis, gastrointestinal disorders, and pulmonary diseases (1, 37). Thus, the ST should be an important tool for the CF diagnosis, particularly in the absence of the identification of mutations in the *CFTR* gene. It has also contributed to deeper understanding of the physiopathology aspects of CF and of the effects of some drugs to restore the function of the CFTR protein. The ST may show controversial results (although rare); therefore, further diagnostic methods are needed in some cases, as we showed in our data, when we identified 10 patients with CF and two *CFTR* mutations belonging to classes I, II, or III, and absence of positive ST.

### Newborn Screening by Immunoreactive Trypsinogen

The implementation of NBS tends to increase the number of CF diagnosis in newborn infants. In CF, the release of trypsinogen in blood is increased as a result of the obstruction caused by secretion accumulation in the pancreatic ducts. High IRT levels are a marker of neonatal screening test for CF (38) and the diagnosis is confirmed by duplicate STs and/or the detection of two mutations in the *CFTR* gene (1).

In our data, the annual number of the ST performed was the same as before and after the implementation of IRT dosage. In addition, we showed a higher number of ST performed in patients aged over 18 years after the implementation of IRT dosage. This fact can be associated with a higher number of solicitations by the adult pneumology, correlated with the improvement of knowledge of (i) CF and residual CFTR function; (ii) presence of *CFTR* mutations included in the classes IV, V, and VI; and (iii) better registry from the adult clinic in the last years.

### Limitations of the Study

Our study shows the following limitations: (i) it did not include a control group, i.e., there may be patients with negative ST with CF; (ii) it was not possible to confirm a CF diagnosis for all patients through genetic studies; (iii) the reason for the ST referral for 1,877/5,277 (35.57%) sweat samples could not be obtained; (vi) there was inclusion of a higher number of subjects under 18 years of age than of older subjects.

### Highlight

(i) High levels of sweat chloride with the presence of CF indicative signs and symptoms are the gold standard for the CF diagnosis.
(ii) Amount of chloride is greater in men than women in all ST reference ranges.
(iii) Indication to perform the ST regarding respiratory symptoms was associated with minor probability of presenting CF disease and age between six months and 18 years.
(iv) Indication to perform the ST regarding digestive symptoms was associated with higher probability of presenting CF disease and age under six months.
(v) CF diagnosis regarding the age cutoffs showed association between them.
(vi) Subjects with digestive symptoms showed higher chloride levels than subjects without this type of symptom. In addition, subjects with respiratory symptoms showed lower chloride levels in their tests than subjects without this type of symptom.
(vii) Indexes [(sweat chloride/sweat sodium) and (sweat sodium–sweat chloride)] used in the article showed association with sex, reason for ST indication, and *CFTR* mutations.
(viii) After the NBS implementation by the IRT, there was a higher chance of borderlines values in the ST and CF diagnosis.
(ix) A wide spectrum of *CFTR* mutation was found with the highest prevalence of F508del identification.
(x) *CFTR* mutation identification or presence of F508del/F508del genotype was associated with higher probability of ST chloride levels ≥60 mEq. Moreover, the absence of *CFTR* mutations identified was associated with borderlines values in the ST, as well as with presence of respiratory symptoms.

### Perspectives

Some issues are not well known in CF disease. For example, a large number of subjects with intermediate amounts of ions in sweat will develop classic forms of CF in the future (39). This fact was observed by Groves et al. (39), who carried out a 15-year monitoring on patients with sweat chloride concentrations between 30 and 59 mEq/L. From all patients with positive NBS by IRT, a positive allele for F508del, and intermediate ST, 14/29 (48%) developed classic CF. These data should be analyzed prospectively in many centers worldwide regarding the values of chloride longitudinally and the inclusion of other ST markers, as we analyzed in the present study (sweat chloride and sweat sodium ratio; sweat sodium–sweat sodium; correlation with sweat chloride and sweat sodium). In addition, the intervals of chloride concentration to perform the ST should be analyzed and revised. The variability in the ST might be associated with the populational structure.

The outlier data from the ST should be considered, taking into account that 2% of US Americans with CF have normal sweat chloride levels in the ST (3, 5). Patients with CF and normal ST should be recognized among the health subjects, or regarding other disease. This fact is more important in the genetic era, when the personalized/precision medicine is available for some *CFTR* mutations and probability will be available to all classes of *CFTR* mutations. The innate variability of ST is associated with variants of the *CFTR* gene, presence of modifier genes, and individual biological variation in the production of chlorides, possibly through other ion channels (3, 30), as long as laboratory errors have not occurred. Furthermore, sweat chloride as a biomarker of CFTR activity in the personalized/precision medicine was already used and should be better understood (40).

The function of CFTR should be better studied. The sweat glands in patients with CF, unlike the glands of healthy individuals, do not excrete sweat in response to beta-adrenergic stimulation, but to cholinergic stimulation. Healthy individuals with one allele with mutation in the *CFTR* gene (e.g., parents of patients with CF) excrete 50% of sweat, due to beta-adrenergic stimulation. Function evaluation tests of the CFTR protein may provide, indirectly, identification of carriers of *CFTR* gene mutations, which enables proper genetic counseling (5, 9). However, this fact is not well studied to be used as daily practice. Other tools could be studied, including the sweat induction by evaporimeter, chloride dosage from saliva, nasal potential difference, rectal biopsies, and organoids so that to evaluate the CFTR presence and function (2–9).

Finally, it is necessary but not sufficient to organize and publish CF diagnosis consensus processes. In addition, monitoring implementation efforts and practices seem essential as recommended in the literature (41–43).

### Conclusion

The ST data showed wide variability, which was dependent on age, sex, reason for examination indication (respiratory and digestive symptoms), *CFTR* mutations, and weight of the sweat sample collected. Sweat sodium concentration is directly correlated with sweat chloride levels and it should be used as a quality parameter.

## Ethics Statement

This study was carried out in accordance with the recommendations of Ethics Committee of the University of Campinas (Protocol no. 474326), with written informed consent from the institution in accordance with the Declaration of Helsinki.

## Author Contributions

AGF, FALM, and JDR made substantial contributions to conception and design, acquisition, analysis, and interpretation of data; they were involved in drafting the manuscript and revising it critically for important intellectual content; gave final approval of the version to be published; and agreed to be accountable for all aspects of the work by ensuring that questions related to the accuracy or integrity of any part of the work have been appropriately investigated and resolved. CCSG, MFS, and AFR made substantial contributions to conception and design of the study, acquisition, analysis, and interpretation of data.

## Conflict of Interest Statement

The authors declare that the research was conducted in the absence of any commercial or financial relationships that could be construed as a potential conflict of interest.

## Abbreviations

CF, cystic fibrosis; CFF, cystic fibrosis foundation; CFTR, cystic fibrosis transmembrane conductance regulator; Cl^−^, chloride ion; IRT, immunoreactive trypsinogen; mEq/L, milliequivalents per liter; MLPA, multiplex ligation-dependent probe amplification; OMIM, online Mendelian inheritance in man; ST, sweat test.
